# Revitalising Brewers' Spent Grains and Enriching With Biogenic Compounds Through the Fermentation of Fructophilic Lactic Acid Bacteria and Yeasts

**DOI:** 10.1111/1751-7915.70171

**Published:** 2025-06-09

**Authors:** Alessandro Stringari, Ali Zein Alabiden Tlais, Stefano Tonini, Daniela Pinto, Giorgia Mondadori, Pasquale Filannino, Raffaella Di Cagno, Marco Gobbetti

**Affiliations:** ^1^ Faculty of Agricultural, Environmental and Food Sciences Free University of Bolzano‐Bozen Bolzano Italy; ^2^ International Center on Food Fermentations Bolzano Italy; ^3^ Human Microbiome Advanced Project Research & Development Milano Italy; ^4^ Department of Soil, Plant and Food Science University of Bari Aldo Moro Bari Italy

**Keywords:** antifungal activity, antioxidant activity, fructophilic lactic acid bacteria, human keratinocytes, wound healing, yeasts

## Abstract

The large output of spent grains from the brewing industry presents environmental concerns but also offers promising nutritional and functional potential for valorization by researchers and industrial stakeholders. In this perspective, we investigated how non‐conventional starters like *Fructobacillus fructosus* PL22 and *Wickerhamomyces anomalus* GY1 can drive the fermentation of brewer's spent grain (BSG), a solid by‐product of the brewing industry, to enrich its portfolio of bioactive compounds. While sugar reduction was comparable between started‐ and unstarted‐BSG, the effect of the fermentation became evident through the release of key microbial metabolites (lactic and acetic acids and ethanol). Both starters generated the highest number of unique peptides, with only one previously identified as antioxidant peptide found in BSG fermented with *F. fructosus* . During fermentation, most amino acids and phenolic compounds decreased, while BSG fermented with *W. anomalus* distinctly enhanced the release of Ala, Cys and GABA, and health‐promoting phenolic compounds, such as gallic acid, gallocatechin, quercetin, naringenin, kaempferol, and isorhamnetin. These metabolic changes were associated with the enhanced antifungal and antioxidant properties, which in turn positively reflected on skin protection as shown by the increased proliferation of human keratinocytes, over‐expression of the filaggrin (*FLG*) gene, and wound healing. The power of fermentation to revitalise BSG, giving it a second life chance through the improvement of its nutritional value and further multifunctionality, was demonstrated.

## Graphical abstract

Non‐conventional starters ( *F. fructosus* PL22 and *W. anomalus* GY1) enrich brewer's spent grain (BSG) portfolio of bioactive compounds. Fermented BSG showed antioxidant and antifungal activities and boosted keratinocyte proliferation, FLG expression, and wound healing.

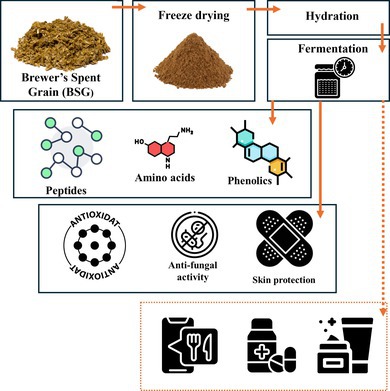

## 1 Introduction

Brewing industry generates various by‐products, with brewer's spent grain (BSG) making up 85% of the total solid by‐products (Zeko‐Pivač et al. 2022). Such by‐product is recognised as a lignocellulosic material rich in fibre, rendering it a nutritious dietary option for animals and a potential resource for soil enrichment (Ribau Teixeira et al. 2020), cultivation of microorganisms and the production of enzymes, or conversion into biofuels (Zeko‐Pivač et al. 2022). Besides, BSG contains up to 30% protein, including hordein, gluten, globulin, and albumin, that are rich in essential amino acids (Wen et al. 2019). Indeed, BSG is also recognised as a suitable alternative to legumes and cereals due to its protein and amino acid composition. The BSG protein is rich in various amino acids, such as glutamine/glutamic acid, proline and leucine, and is suggested to integrate with conventional food (Shroti and Saini 2022). Polyphenols, another crucial component in BSG, have been associated with several health‐promoting effects (Macias‐Garbett et al. 2021).

These multifaceted components have prompted recent interest in utilising BSG as an ingredient in fortifying foods, designing pharmaceutical formulations, and developing cosmetic preparations (Xiros and Christakopoulos 2012). Despite these advantages, some limitations still persist. For instance, even after extraction and brewing, a significant protein fraction remains insoluble (Abeynayake et al. 2022), and consequently, proteins require a preliminary proteolysis to fully demonstrate their potential in developing new protein‐related products, while phenolics bound to the ligninocellulosic structure of BSG are poorly bioaccessible (Verni et al. 2020). Challenges also arise from sensory alteration, impacting colour, aroma and physical properties, necessitating the use of only small amounts (5%–10%) in foods (Johnson et al. 2010). To address the limitations and unlock the latent value of this low‐cost resource, bioprocessing, particularly fermentation, has proven to be a viable strategy.

Fermentation is renowned for enhancing the functionality of products or generating new ones with multitask capabilities (Tlais et al. 2023). During fermentation, endogenous and microbial proteases synergically break down proteins, releasing peptides and hydrolysates with demonstrated bioactivities, such as antioxidant, antifungal, anti‐inflammatory and angiotensin‐I‐converting enzyme inhibitory properties (Verni et al. 2020). Additionally, hydrolytic enzymes, like xylanase, β‐glucanase, α‐amylase, cellulase, esterase, oxidase and glucosidase, improve the bioavailability and bioaccessibility of phenolic compounds as well as the generation of new derivatives, leading to changes in colour, flavour and texture (Tlais et al. 2022). Lactic acid fermentation has proven to be an efficient option for enhancing the nutritional and sensory properties of pasta fortified with BSG (Neylon et al. 2021a) or improving the crumb hardness, specific volume and sensory profiling of bread made with fermented BSG (Neylon et al. 2021b). The inclusion of fermented BSG enhanced the nutritional and antioxidant properties of biscuits, as well as positively influenced the in vitro digestion and glucose release (Wang et al. 2023). When applied in mayonnaise formulations, the fermented hydrolysate demonstrated superior emulsion stability (Chin et al. 2022).

Research also recognised the skin‐lightening properties of BSG bioactive compounds in cosmetics preparations (Macias‐Garbett et al. 2021) as well as the ability to attenuate melanin synthesis by inhibiting tyrosinase (Almendinger et al. 2020). Despite this potential, practical applications remain limited, with only a few commercialised products, such as BSG wax and Barley TONIQ. The permeability of BSG components in human skin layers has also not been thoroughly investigated (Macias‐Garbett et al. 2021).

Despite this extensive research on BSG fermentation, there remains ample scope for further exploration due to the vast diversity of microorganisms possessing varied enzyme profiles (Filannino et al. 2018). Building upon this premise, this study explored the metabolic potential of non‐conventional fructophilic lactic acid bacteria (FLAB) ( *Fructobacillus fructosus* ) and yeast (*Wickerhamomyces anomalus*) that have not been previously employed for BSG fermentation. Generally, *Fructobacillus* spp. are known for their metabolic capability to modify plant secondary metabolites, such as alkaloids and phenolic acids (Filannino et al. 2016). In addition to their documented antimicrobial, antifungal and probiotic properties (Patil et al. 2020; De Simone et al. 2023), emerging evidence suggests that *F. fructosus* may enhance food functionality by modulating the gut microbiota, improving host antioxidant status and potentially contributing to immune regulation. *Wickerhamomyces anomalus* is a metabolically versatile yeast capable of producing β‐glucosidase, esterase, polygalacturonase and proteolytic enzymes (Tonini et al. 2024; Martos et al. 2013). Beyond its ecological resilience (Padilla et al. 2018), *W. anomalus* is notable for synthesising antifungal compounds via diverse metabolic routes, supporting its application as a biocontrol agent and natural preservative in cereal‐based products (Coda et al. 2011; Lanhuang et al. 2022). Additionally, its ability to enrich the profile of flower‐based formulations has been demonstrated, thereby enhancing their antioxidant capacity (Tonini et al. 2024).

In this study, we aimed to investigate the capability of *F. fructosus* and *W. anomalus* in augmenting the profiling of bioactive compounds (phenolic compounds, amino acids and bioactive peptides) of BSG. Thereafter, we intended to establish possible associations between these BSG modifications and ex vivo protective effects versus human keratinocyte cell line. Promising prospects of fermented BSG in potential applications in dermatological and cosmetic formulations are proposed.

## 2 Experimental Procedure

### 2.1 Plant Materials, Microorganisms and Culture Conditions

The BSG was provided by Birra Forst S.p.a. (Lagundo, Italy). Samples were transported to the Micro4Food laboratory of the Faculty of Agricultural, Environmental and Food Sciences, Libera Università di Bolzano, Bolzano, Italy, where they underwent immediate freeze‐drying using the Epsilon 2‐6D LSC plus freeze‐drier (Martin Christ, Osterode am Harz, Germany) and were stored under refrigerated conditions until further use. *Fructobacillus fructosus* PL22, isolated from bee‐collected pollen (Di Cagno et al. 2019) and *Wickerhamomyces anomalus* GY1, isolated from *Malus domestica* cultivar Golden Delicious (personal data), were used as starter cultures. Both microbial strains belong to the Culture Collection of the Micro4Food laboratory. Before fermentation experiments, FLAB and yeast cultures were maintained as stocks in 20% (v/v) glycerol at −20°C and routinely propagated at 30°C for 24 h in Fructose Yeast extract Peptone (FYP) and Sabouraud Dextrose broths, respectively.

### 2.2 Fermentation of BSG

Freeze‐dried BSG was rehydrated with 90% of sterile distilled water and subsequently homogenised with a Classic Blender (MB 550, Microtron, Italy) to obtain a liquid suspension. Cells were propagated in their growth medium until reaching their late exponential growth phase (ca. 18–22 h). Then, cells were harvested through centrifugation (10,000 × *g*, 10 min at 4°C), washed twice in sterile physiological solution (NaCl 0.9%, w/v) and singly re‐suspended into BSG homogenate to a final cell density corresponding to ca. 7.0 and 5.0 Log CFU/g for FLAB and yeast, respectively. The BSG homogenate was subjected to batch fermentation in duplicate using sealed glass vessels under static conditions at 30°C for 72 h, resulting in samples named Fermented‐BSG. The BSG without microbial inoculum and incubation (Raw‐BSG) and BSG without inoculum but incubated at 30°C (Unstarted‐BSG) were used as the controls.

### 2.3 Microbial Growth and Acidification

To monitor kinetic growth and acidification, samples were taken at the beginning of the fermentation (T0) and after 24 (T24), 48 (T48), and 72 (T72) h of fermentation. The BSG samples (10 mL) were homogenised in sterile 0.9% (w/v) NaCl using a Stomacher 400 lab blender (Seward Medical). Serially diluted aliquots were plated on specific agar media. FLAB and yeasts were enumerated on FYP agar containing 0.1% of cycloheximide (Sigma Chemical Co.) at 30°C for 48 and 72 h under anaerobiosis and on Sabouraud Dextrose agar (SDA, Oxoid) with 150 ppm of chloramphenicol at 30°C for 72 h, respectively. A pH‐meter (SensION+ PH3, Hach, Italy) equipped with a food penetration probe was used for pH measurement. The monitoring of the starters after the fermentation was carried out by Random Amplified Polymorphic DNA‐Polymerase Chain Reaction (RAPD‐PCR) using P4 primer for lactic acid bacteria (LAB) and mM13 for yeasts (De Angelis et al. 2001; Del Bove et al. 2009).

### 2.4 Carbohydrates, Organic Acids and Ethanol Quantification

Carbohydrate consumption and the synthesis of organic acids and ethanol were assessed in water‐soluble extracts (WSE) prepared from Raw‐, Unstarted‐ and Fermented‐BSG samples. One gram of each sample was homogenised with 9 mL of Tris–HCl (0.1 mM, pH 8.8) and incubated at 4°C for 1 h with constant stirring (200 rpm). After incubation, the mixture underwent centrifugation at 12,000 × *g* for 10 min. The resulting WSE was filtered through a 0.22‐μm membrane filter (Millipore Corporation) and stored at −20°C until further analysis. Quantification of maltose, glucose, fructose, mannitol, lactic acid, acetic acid and ethanol was performed using high performance liquid chromatograph (HPLC) equipped with an Aminex HPX‐87H column (ion exclusion, Biorad), a Perkin Elmer 200a refractive index detector (RI), and a UV detector operating at 210 nm (Tlais et al. 2022). Standards for carbohydrates, organic acids and ethanol were purchased from Sigma‐Aldrich (Steinheim, Germany).

### 2.5 Total Protein Quantification

Total nitrogen content of Raw‐, Unstarted‐ and Fermented‐BSG samples was measured by the NDA 702 Dumas Nitrogen Analyser (VELP Scientifica 2014). Freeze‐dried samples were weighed and carefully wrapped in tin foil before the analysis. Based on the nature of the sample, oxygen dosage was fixed at a rate of 400 mL/min to achieve the best combustion. Total nitrogen results were obtained using VELP DUMASoftTM 6.1.0 and converted to total protein content by multiplying by the standard conversion factor 6.25 (Rhee 2001).

### 2.6 Peptides and Amino Acids Quantification and Profiling

Peptide concentration in WSE was determined by the *o*‐phthalaldehyde (OPA) assay (Church et al. 1985). Peptide profiles were analysed by reversed‐phase fast performance liquid chromatography (RP‐FPLC), using a Resource RPC column and “AKTA FPLC equipment”, with the UV detector operating at 214 nm (GE Healthcare Bio‐Sciences AB, Uppsala, Sweden) as previously described by (Pontonio et al. 2020). Total and individual free amino acids in WSE were analysed by a Biochrom 30 series Amino Acid Analyser (Biochrom Ltd., Cambridge Science Park, England) with a Li‐cation‐exchange column (20 × 0.46 cm inner diameter) as described by (Rizzello et al. 2010). Briefly, proteins and peptides were precipitated by the addition of 5% (v/v) cold solid sulfosalicylic acid containing 500 μmol/L of L‐Norleucine as internal standard, holding at 4°C for 1 h and centrifuging at 10,000 rpm for 5 min. The supernatant was filtered again through a 0.22‐μm pore size filter. Amino acids were post column derivatized with ninhydrin reagent and detected by absorbance at 440 (proline) or 570 nm (all the other amino acids).

### 2.7 Identification of Low Molecular Weight Peptides by UHPLC/HRMS2

To separate the active peptides fraction, the WSE underwent ultrafiltration (molecular weight cut‐off < 3 kDa), following a method adapted from (Tagliazucchi et al. 2017) with minor modifications. In this procedure, 15 mL of the sample was loaded into a Vivaspin20 column with a 3000 MWCO‐PES membrane (Sartorius, Italy) and centrifuged at 6000 rpm for 80 min to obtain low‐molecular‐weight water‐soluble peptide extract (LMW‐WSE). Low molecular weight peptides were identified through UHPLC/HR‐MS2 (UHPLC Ultimate 3000, Thermo Scientific, San Jose, CA, USA; Q Exactive Hybrid Quadrupole‐Orbitrap Mass Spectrometer, Thermo Scientific, San Jose, CA, USA) equipped with a C18 column (Acquity UPLC‐C18 Reversed‐phase, 2.1 × 100 mm, 1.8 μm particle size, Waters Corporation, Milford, MA, USA). MS data analysis was processed using Proteome Discoverer 2.3 (Thermo Fisher Scientific, Dreieich, Germany) coupled with Matrix software (Matrix Science, Boston, MA, USA) for peptide sequencing and identification. Key parameters for the identification process included: enzyme, no‐enzyme; peptide mass tolerance, ± 5 ppm; fragment mass tolerance, ± 0.1 Da; variable modification, Demetilation (NQ), oxidation (M) and phosphorylation (ST); the maximal number of post‐translational modifications permitted in a single peptide, 1. Peptide and protein identification results were exported after filtering with the Peptide and Protein Validator to achieve a false discovery rate (FDR) below 0.01. Identified peptides were compared with bioactive peptides documented in the literature using the Bioactive Peptide Database BIOPEP UWM (Minkiewicz et al. 2019).

### 2.8 Identification and Quantification of Free Phenolic Compounds

To assess the impact of fermentation on phenolic composition, profiling of free phenolic compounds in methanol/water‐soluble extract (MWSE) was carried out using an LC–MS platform composed of an Ultimate 3000 RSLCnano system, coupled to a QExactive Plus Hybrid Quadrupole‐Orbitrap mass spectrometer (Thermo Fisher Scientific, Waltham, MA, USA). The MWSE was derived from Raw‐, Unstarted‐ and Fermented‐BSG samples. The extraction process involved mixing 1 g of the sample with 5 mL of a methanol/water solution (80:20, v:v) acidified with hydrochloric acid (0.1%, v/v). Sonication was performed for 1 min (two cycles, 30 s/cycle, 5 min interval between cycles) in an ice‐bath, followed by an additional hour of extraction under stirring conditions at room temperature. A Waters Acquity HSS T3 column (1.8 μm, 100 mm × 2.1 mm) (Milford, MA, USA) was used for chromatographic separations of phenolic compounds, applying the following binary gradient program: 0 min, 2% B; from 0 to 3 min, linear gradient to 20% B; from 3 to 4.3 min, isocratic 20% B; from 4.3 to 9 min, linear gradient to 45% B; from 9 to 11 min, linear gradient to 100% B; from 11 to 13 min, wash at 100% B; from 13.01 to 15 min, back to the initial conditions of 5% B (solvent A = water +0.1% formic acid; solvent B = acetonitrile +0.1% formic acid). The flow rate was 300 μL/min, and the temperature of the column was set at 40°C. The LC–MS system was equipped with a Higher Collisional Energy Dissociation cell (HCD) and a HESI (Heated Electro Spray Ionisation) interface was adopted for LC‐HRMS coupling. The MS detection following chromatographic separation was performed in Targeted‐SIM/dd‐MS^2^ mode and in negative polarity using the following parameters: spray voltage, 2.80 kV; sheath gas flow rate at 35 arbitrary units; auxiliary gas flow rate at 10 arbitrary units; capillary temperature at 300°C; S lens RF level at 50 arbitrary units; capillary gas heater temperature, 280°C. The settings for the Q‐ExactiveTM mass spectrometer were the following: mass scan range, 80–1200 m/z; resolution, 70.000 (FWHM); Automatic Gain Control (AGC) Target, 5 × 10^6^ ions; maximum injection time (IT), 100 ms. Compounds were identified based on their reference standard, retention time and [M− H]^−^ ions. Peak areas, obtained from eXtracted Ion Current (XIC) chromatographic traces, that is, chromatograms created by extracting the ion current from HRMS spectra in a m/z interval, including the monoisotopic peak, were used as a measurement of MS response. They were thus employed to perform calibrations based on commercial standards acids and, afterwards, for the quantification of these phenolic compounds in BSG extracts. The Xcalibur v. 3.1 software (Thermo Fisher Scientific, Waltham, MA, USA) was used for the control of the Q‐Exactive plus spectrometer and for data elaboration.

### 2.9 In Vitro Antifungal and Antioxidant Activities

Hyphal radial growth rate assay was employed to evaluate the in vitro antifungal activity of MWSE and LMW‐WSE, as described by (Rizzello et al. 2011) with some modifications. The extracts were incorporated into mini‐Petri dishes, and Potato Dextrose Agar (PDA) (Oxoid Ltd., Basingstoke, Hampshire, England) medium was subsequently poured over, ensuring uniform distribution of the extract within the growth media. *Aspergillus versicolor* CBS 117286, *Penicillium roqueforti* DPPMA1 and *Penicillium carneum* CBS 112297 were chosen as mould indicators. A 5 mm Ø fresh fungal mycelium from the refreshed culture was placed in the center of the PDA/LMW‐WSE or MWSE mini‐plates, serving as the inoculum for the antifungal assay. After 5 days of incubation at room temperature, the hyphal radial growth rate was measured, and the corresponding inhibition percentage was calculated using the formula: Growth inhibition (%) = [(mycelial growth on PDA − mycelial growth on PDA/LMW‐WSE or MWSE/mycelial growth on PDA)] × 100.

The LMW‐WSE and MWSE were used to determine the antioxidant activity. The DPPH radical scavenging activity was measured by using 1,1‐diphenyl‐2‐picrylhydrazyl radical (DPPH), as previously described by (Yu et al. 2003). The absorbance values of an antioxidant standard [75 mg/L butylated hydroxytoluene (BHT)] were used to estimate the radical scavenging ability of the BSG extracts. ABTS radical scavenging activity was evaluated by the Antioxidant Assay kit (Sigma‐Aldrich) according to the manufacturer's instructions. This assay involves the formation of a ferryl myoglobin radical from metmyoglobin and hydrogen peroxide, leading to the oxidation of ABTS (2,20‐azino‐bis(3‐ethylbenzothiazoline‐6‐sulphonic acid)) and the production of a green‐coloured radical cation, ABTS^+^, detectable spectrophotometrically at 405 nm. Trolox, a water‐soluble vitamin E analog, was used as a control antioxidant.

### 2.10 Human Keratinocyte Cell Culture

Normal human keratinocyte NCTC 2544 cells (HL97002) were supplied by Istituto Nazionale di Ricerca sul Cancro (Italy). NCTC 2544 cells were cultured in RPMI 1640 medium, supplemented with 10% fetal bovine serum (FBS), 2 mM L‐glutamine, penicillin (100 U/mL)/streptomycin (100 U/mL) and 50 mg/mL gentamicin (Perry et al. 1957). NCTC 2544 cells were maintained in 25 cm^2^ sterile culture flasks, incubated at 37°C in a humidified atmosphere with 5% CO_2_. When ca. 80% confluence was reached, cells were harvested with trypsin/EDTA and seeded at a density of 5 × 10^4^ cells per well into 96 well plates for 3‐(4,5‐dimethylthiazol‐2‐yl)‐2,5‐diphenyltetrazolium bromide proliferation (MTT) assay, or at a density of 1 × 10^6^ cells per well into 12 well plates for wound scratch assays and RNA extraction.

### 2.11 Viability Assay

The MTT assay was carried out according to the method described by Mosmann (1983) with slight modifications. Briefly, NCTC 2544 were seeded in 96‐well plates at a density of 5 × 10^4^ cells per well and incubated at 37°C, 5% CO_2_ until reaching ca. 80% confluency. Subsequently, the medium was removed from the wells, and the cells were incubated with freeze‐dried samples diluted in RPMI 1640 medium at the following concentrations: 0.05, 0.1, 0.5, 1, 5 and 10 mg/mL. Untreated cells were used as the control. Cells were then incubated for 24, 48 and 72 h at 37°C, and 5% of CO_2_. After the incubation, the medium was removed, and 100 μL of MTT reagent was applied to the cells (stock solution 5 mg/mL in DPBS, diluted 1:10 in RPMI 1640 medium without phenol‐red). The plate was incubated for 3 h under dark conditions at 37°C, and 5% of CO_2_. Then, the MTT solution was aspirated, and 100 μL of dimethyl sulphoxide (DMSO) was added to dissolve the purple formazan product. The solution was shaken in the dark for 15 min at room temperature. The absorbance of the solutions was read at 570 nm in a microplate reader (BioTek Instruments Inc., Bad Friedrichshall, Germany). Data were expressed as cell viability percentage compared to the control cells, as per the following formula: % cell viability/ctrl = (Abs sample/Abs ctrl) × 100.

### 2.12 Wound Scratch Assays

Sub‐confluent monolayers of NCTC 2544 cells seeded in 12‐well plates were scratched with a sterile P200 pipette tip. After removal of debris through two washing steps in PBS, cells were exposed to freeze‐dried BSG samples diluted in basal serum‐free medium at a concentration of 0.5 and 0.1 mg/mL. Hyaluronic acid (2 μg/mL) was also used. Basal serum‐free medium was used as the control. Plates were incubated at 37°C, under a 5% CO_2_ atmosphere. Images of three different zones of each monolayer were taken using a phase‐contrast Leica DMIL microscope (Leica Microsystems Srl, Heerbrugg, Switzerland) with a 10× objective. Images were acquired from the same field at 0, 6, 24 and 30 h post‐scratching. Images were analysed using Leica Application Suite software v. 2.8.1 (Leica Microsystems Srl, Heerbrugg, Switzerland). Data were acquired in the form of scratch area (open area) percentage by using Tscratch software (Gebäck et al. 2009). For each scratch experiment, the percentage of the starting scratch area with respect to time 0 and normalised to the control were determined (Pinto et al. 2011).

### 2.13 RNA Extraction and Real‐Time‐PCR on Filaggrin Gene

Gene expression of Filaggrin (*FLG*) in NCTC 2544 cell line was evaluated through quantitative reverse transcription‐ polymerase chain reaction (qRT‐PCR). After reaching 80% confluency, cells were incubated for 24 and 48 h with freeze‐dried BSG samples at 0.1 and 0.5 mg/mL in RPMI 1640 supplemented medium. Untreated cells were used as control. At the end of treatment, total RNA was extracted from NCTC 2544 according to the method described by Chomczynski and Mackey 1995. Complementary DNA (cDNA) was synthesised by reverse transcriptase using commercial kit “PrimeScriptTM RT Reagent Kit (perfect Real Time)” (TakaraBioInc., Japan). Produced amplicons were quantified through the monitoring of the fluorescence emitted during reaction by using the TaqMan (AppliedBiosystems) probes system. The following TaqMan probes were used: Hs00863478_g1 (*FLG*), and Hs99999905_m1 (*GAPDH*) as housekeeping gene. The obtained data were analysed according to the 2^−ΔΔCt^ method (Vigetti et al. 2008).

### 2.14 Statistical Analysis

All analyses were carried out in triplicate for two batches of samples. Most data were subjected to one‐way or two‐way analysis of variance (ANOVA) within the framework of the Generalised Linear Model (GLM). The data were tested for normality and homogeneity of variance assumptions using the Shapiro–Wilk test on residuals and Levene's Test, respectively. In cases where the assumption of homoscedasticity was violated, the reliability of standard ANOVA was limited. Consequently, the GLM was employed to determine statistical differences among group means, offering a more robust alternative due to its flexibility in incorporating interaction effects and managing variance heterogeneity. Pairwise comparison of treatment means was achieved by Tukey‐adjusted comparison procedure with *p*‐value < 0.05. Data from MTT assay, wound scratch assay and qRT‐PCR were analysed using Student's t‐test, with statistical significance set at *p* < 0.05.

## 3 Results

### 3.1 BSG Fermentation

**FIGURE 1:**
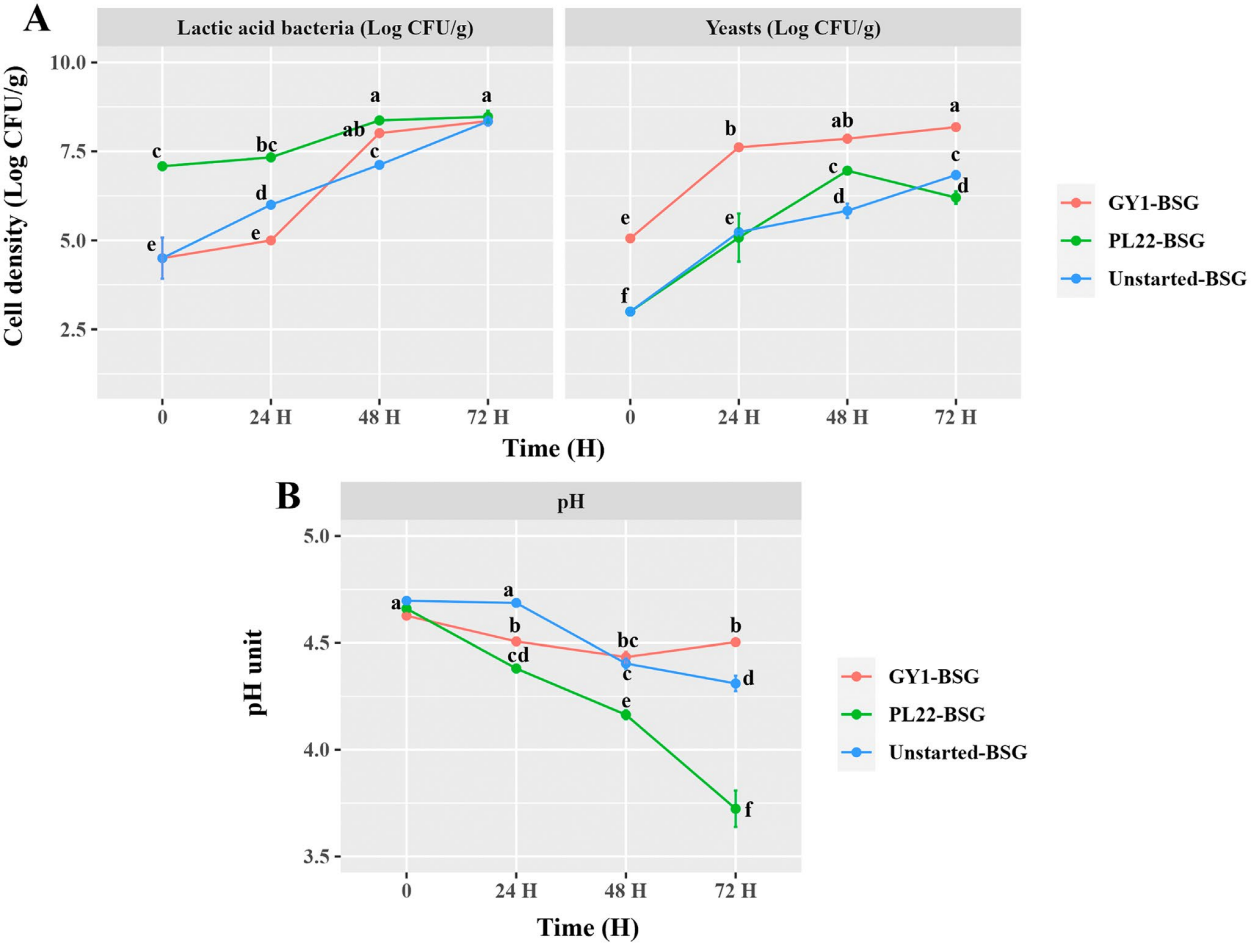
Cell density of presumptive lactic acid bacteria and yeasts (A) and kinetics of pH (B) of brewer's spent grain (BSG) singly fermented at 30°C for 72 h with *Fructobacillus fructosus* PL22 (PL22‐BSG) and *Wickerhamomyces anomalus* GY1 (GY1‐BSG). BSG incubated under the same conditions, except for the use of starters, was used as the control (Unstarted‐BSG). Data represent the mean ± standard deviation of two biological replicates (each analysed in technical triplicate). Data points with different superscript letters differ significantly (*p* < 0.05).

Following the implementation of a protocol involving freeze‐drying, rehydration and the inoculum of starters (ca. 7 Log CFU/g for PL22 and ca. 5 Log CFU/g of GY1), the fermentation of BSG was conducted at 30°C for 72 h. Before the fermentation, as estimated by plating on FYP and SDA agar media, the endogenous cell density of presumptive FLAB and yeasts in BSG was 4.50 ± 0.50 and 3.00 ± 0.00 Log CFU/g, respectively (Figure 1A). BSG fermented with FLAB (PL22‐BSG) reached the highest significant (*p* < 0.05) cell number of presumptive FLAB after 48 h (8.37 ± 0.05 Log CFU/g), while GY1‐BSG and Unstarted‐BSG required an additional 24 h to attain a similar value. Likewise, an upward trend in yeast counts was observed throughout the fermentation, with GY1‐BSG consistently showing the higher significant (*p* < 0.05) count compared to other samples (Figure 1A). After fermentation, RAPD‐PCR biotyping confirmed the presence of *F. fructosus* PL22 in PL22‐BSG, in contrast to the distinct profiles of autochthonous LAB observed in Raw‐BSG, Unstarted‐BSG and GY1‐BSG. Similarly, *W. anomalus* GY1 was uniquely identified in GY1‐BSG. The yeast RAPD‐PCR profile in PL22‐BSG was similar to that of the isolates from Unstarted‐BSG, but different from that of the Raw‐BSG (Figure S1). The observed microbial growth reflected the decline in pH across the fermented samples, starting from an initial pH value in Raw‐BSG of 4.71 ± 0.00. As expected, the most significant (*p* < 0.05) pH reduction was found after 72 h of fermentation in PL22‐BSG (3.72 ± 0.07), followed by Unstarted‐BSG (4.31 ± 0.03) and GY1‐BSG (4.50 ± 0.01) (Figure 1B).

### 3.2 Carbohydrates, Organic Acids and Ethanol Quantification

**FIGURE 2:**
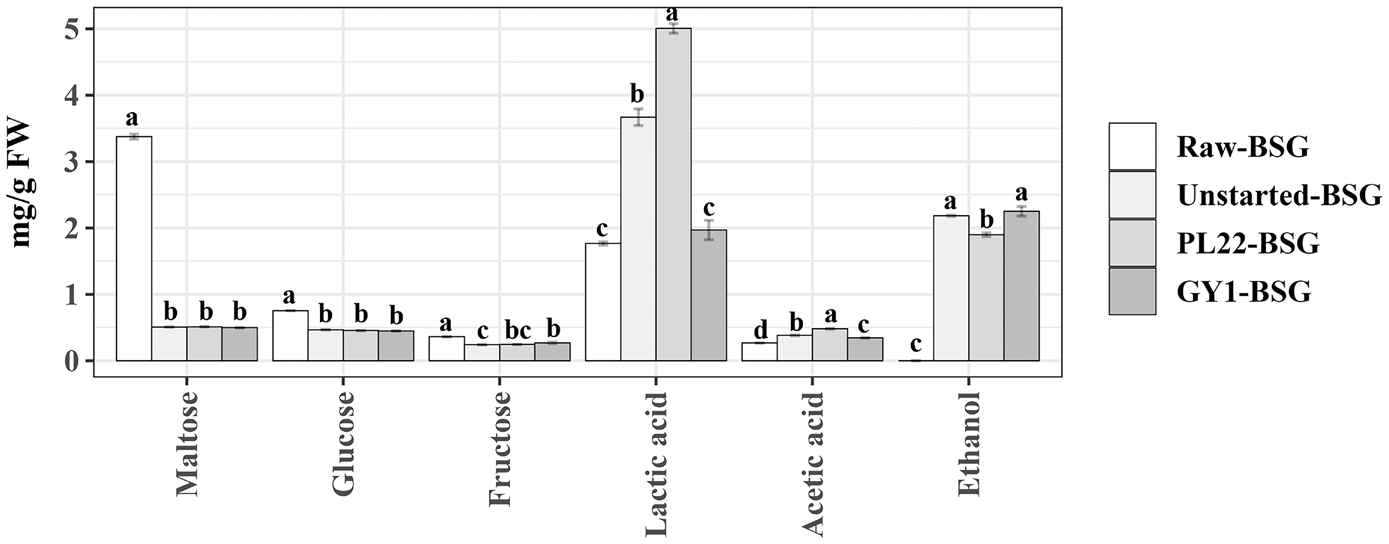
Carbohydrates, organic acids and ethanol quantification (mg/g FW) in raw brewer's spent grain (Raw‐BSG) and BSG fermented with *Fructobacillus fructosus* PL22 (PL22‐BSG) and *Wickerhamomyces anomalus* GY1 (GY1‐BSG). Fermentation was carried out for 72 h at 30°C. BSG incubated under the same conditions, except for the use of starters, was used as the control (Unstarted‐BSG). Data represent the mean ± standard deviation of two biological replicates (each analysed in technical triplicate). Bars with different superscript letters differ significantly (*p* < 0.05).

As expected, maltose was the most dominant carbohydrate of BSG (3.37 ± 0.03 mg/g Fresh weight (FW)), followed by glucose (0.75 ± 0.01 mg/g FW) and fructose (0.36 ± 0.01 mg/g FW) (Figure 2). After fermentation, a noteworthy (*p* < 0.05) consumption of maltose occurred across Unstarted‐BSG and Started‐BSG samples. A decreasing trend was also found for glucose and fructose concentrations. As expected, the main microbial metabolites identified were lactic acid, acetic acid and ethanol. The synthesis of lactic acid was found in BSG fermented with *F. fructosus* PL22, showing the highest concentration, followed by Unstarted‐BSG. Similarly, BSG fermented with the FLAB PL22 showed the highest acetic acid concentration. Although the production of ethanol was found across all samples, Unstarted‐BSG and especially GY1‐BSG had the highest significant (*p* < 0.05) values.

### 3.3 Total Proteins and Peptides Profiling

Protein and peptide concentrations in Raw‐BSG were 250.5 ± 0.33 and 4.78 ± 0.09 mg/g dry weight (DW), respectively (Figure S2). Compared to that in the Raw‐BSG, only the PL22‐BSG sample showed a significant increase (*p* < 0.05) in protein concentrations (282.9 ± 16.03 mg/g DW). In contrast, the most pronounced (*p* < 0.05) release of peptides occurred in Unstarted‐BSG (6.84 ± 0.04 mg/g DW) and GY1‐BSG (7.04 ± 0.10 mg/g DW), and to a lesser extent in PL22‐BSG (6.09 ± 0.13 mg/g DW). The chromatographic analyses distinctly revealed a higher modified peptide profile in Unstarted‐ and Fermented‐BSG samples compared to that in Raw‐BSG, as highlighted by the generation of peaks with differing intensities and characteristics (Figure S3).

### 3.4 Peptidomic Analysis

**FIGURE 3:**
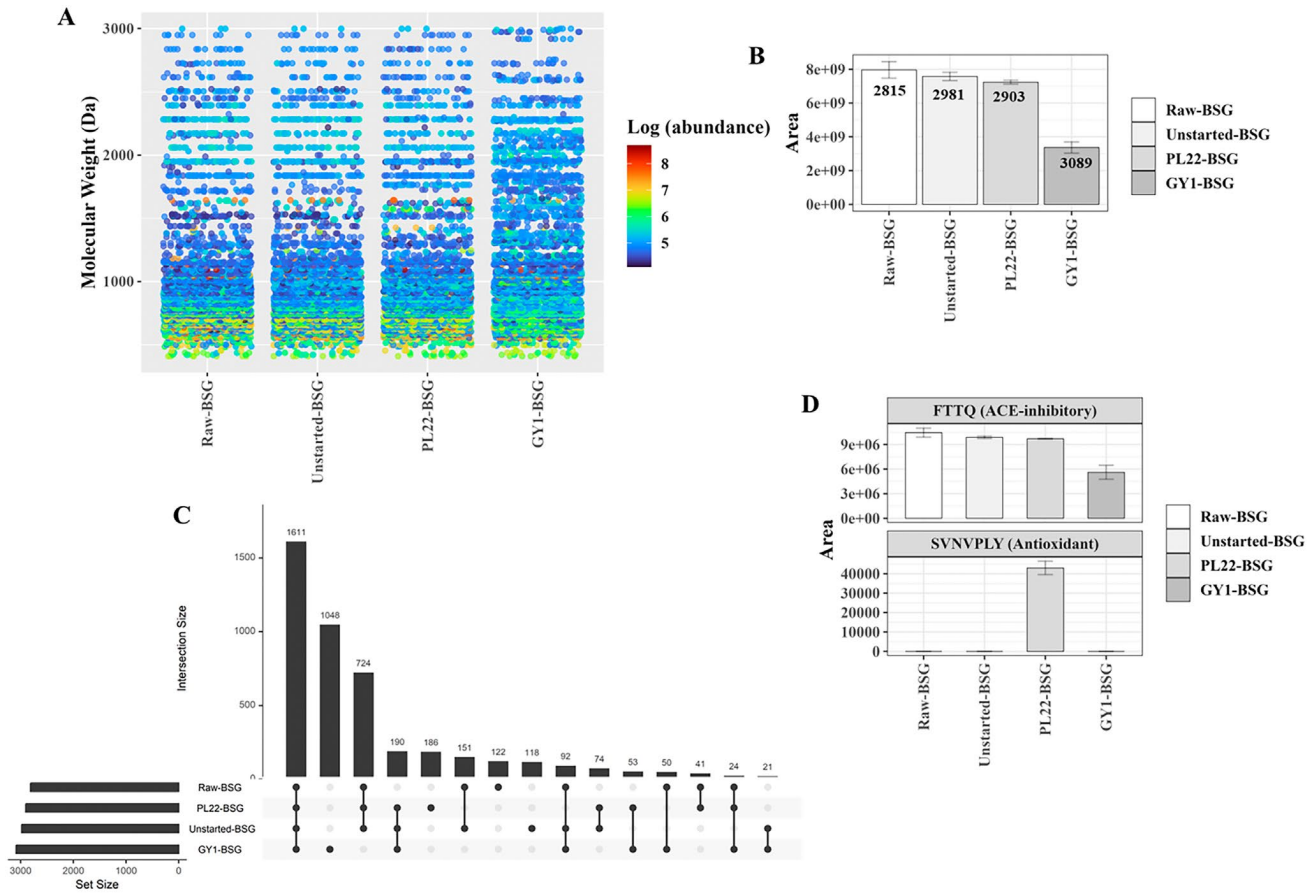
Peptidomic analyses of low molecular weight water soluble extracts (LMW‐WSE) obtained from raw brewer's spent grain (Raw‐BSG) and BSG fermented with *Fructobacillus fructosus* PL22 (PL22‐BSG) and *Wickerhamomyces anomalus* GY1 (GY1‐BSG). Fermentation was carried out for 72 h at 30°C. BSG incubated under the same conditions, except for the use of starters, was used as a control (Unstarted‐BSG). The distribution of identified peptides based on the molecular weight, employing a colour scale that transitions from blue to red to represent the Log abundance of each identified peptide within each sample (A); total number and abundance of different peptides found in each sample (B); upset plot of the intersection of samples, sorted by identified peptides, (dark circles in the matrix indicate sets that are part of the intersection) (C); abundance of bioactive peptides found in each sample (D).

To separate the active peptide fraction, WSE underwent ultrafiltration to yield LMW‐WSE. High‐Resolution Mass Spectrometry (HRMS) was employed for comprehensive characterisation of peptide compositions of the extracts. The untargeted approach resulted in divergence from colorimetric findings but provided valuable insights into the impact of fermentation on peptide generation, nature, distribution and composition. The identified peptides, ranging from 4 to 32 amino acids, revealed sequences associated with parental proteins from *Hordeum vulgare* . Molecular weight (MW) distribution, as ascertained through Label‐Free Quantification (LFQ) analysis, ranged ca. from 400 to 3000 Da (Figure 3). A total of 4505 peptides were revealed among all samples, with only 2815 peptides identified in Raw‐BSG. After the incubation, a conspicuous increase in the diversity of peptides was observed compared to that in Raw‐BSG, especially in GY1‐BSG (3089), notwithstanding its comparatively lower cumulative abundance of identified peptides (Figure 3). Fermentation played a pivotal role in the metabolic dynamics of peptides, contributing to the enhancement of specific peptide abundances, the hydrolysis of others and the generation of entirely new peptides. Both starters induced the production of unique peptides, with GY1‐BSG generating 1048 and PL22‐BSG generating 190 peptides. A cross‐referencing of identified peptides against established bioactive peptides (BPs) through the BIOPEP UWM database revealed that only two of the total identified peptide sequences displayed 100% sequence homology with previously recognised and validated BPs. In a semi‐quantitative assessment, the presence of FTTQ, recognised for its ACE‐inhibitory attributes, was evident across all samples, albeit with the least abundance observed in GY1‐BSG. Conversely, SVNVPLY, associated with antioxidant activity, was exclusively found in PL22‐BSG (Figure 3).

### 3.5 Amino Acids Profiling

The fermentation significantly (*p* < 0.05) changed the free individual amino acid composition of BSG. When considering total free amino acid concentration, only GY1‐BSG showed a noteworthy reduction (ca. 33%) (*p* < 0.05) compared to that in Raw‐BSG (1070.08 ± 18.98 mg/kg DW). Pro, Leu, Ala, Arg and Lys were identified as the most abundant amino acids in Raw‐BSG (Table 1). Notable changes were observed in individual free amino acid concentrations, reflecting the dynamic effect of fermentation processes. For instance, several amino acids (Thr, Ile, Leu, Tyr) were markedly (*p* < 0.05) reduced in Unstarted‐BSG and PL22‐BSG and to a higher extent in GY1‐BSG. Ser and Gln underwent complete metabolism in Unstarted‐BSG and PL22‐BSG, while GY1‐BSG showed comparable (*p* > 0.05) values to those in Raw‐BSG for Ser and lower (*p* < 0.05) concentrations for Gln. Phenylalanine (Phe) diminished in all samples, while Arg was completely metabolised in Unstarted‐BSG and GY1‐BSG, with low concentrations found in PL22‐BSG. Compared to that in Raw‐BSG, Asp and Glu were significantly synthesised only in Unstarted‐BSG and PL22‐BSG, while Ala, Cys and GABA in GY1‐BSG, and Pro in PL22‐BSG (Table 1).

**TABLE 1:** Quantification of the individual and total free amino acids (TFA) (mg/kg DW) in raw brewer's spent grain (Raw‐BSG) and BSG fermented with *Fructobacillus fructosus* PL22 (PL22‐BSG) and *Wickerhamomyces anomalus* GY1 (GY1‐BSG).

Samples	Raw‐BSG	Unstarted‐BSG	PL22‐BSG	GY1‐BSG
Asp	18.2 ± 0.5^b^	27.1 ± 1^a^	26.2 ± 0.1^a^	7.5 ± 0.9^c^
Thr	30.3 ± 0.2^a^	15.1 ± 1.7^b^	14.6 ± 0.7^b^	6.9 ± 0.9^c^
Ser	11.0 ± 0.4	n.d.	n.d.	14.4 ± 3.0
Glu	52.9 ± 0.4^b^	190.3 ± 31.5^a^	180.2 ± 9.5^a^	24.4 ± 0.1^b^
Gln	74.7 ± 1.7^a^	n.d.	n.d.	64.6 ± 0.6^b^
Ala	94.6 ± 0.4^b^	110.3 ± 2.2^b^	69.4 ± 5.5^c^	192.0 ± 5.0^a^
Val	77.2 ± 1.1^a^	57.7 ± 6.7^a^	32 ± 2.9^b^	21.0 ± 0.7^b^
Cys	n.d.	n.d.	n.d.	10.6 ± 2.3
Met	4.3 ± 2^ab^	0.9 ± 0.2^b^	7.2 ± 0.9^a^	0.2 ± 0.1^b^
Ile	42.0 ± 1.4^a^	26.7 ± 2.4^b^	19.1 ± 0.7^b^	6.0 ± 0.1^c^
Leu	128.2 ± 0.9^a^	69.6 ± 2.4^b^	39.6 ± 4^b^	9.5 ± 1.5^c^
Tyr	54.2 ± 9.3^a^	10.8 ± 0.1^b^	1.7 ± 0.6^b^	n.d.
Phe	41.7 ± 0.4	n.d.	n.d.	n.d.
GABA	46.2 ± 0.9^b^	37.4 ± 1.3^ab^	52.6 ± 6.7^ab^	60.6 ± 1.3^a^
Lys	80.4 ± 1.6^a^	42.8 ± 2.5^b^	19.3 ± 0.4^c^	26.6 ± 1.9^c^
Arg	89.5 ± 0.6^a^	n.d.	3.5 ± 0.1^b^	n.d.
Pro	224.8 ± 2.3^b^	295.3 ± 16.5^b^	429.8 ± 17.6^a^	282.1 ± 4.8^b^
TFA	1070.1 ± 19.0^a^	883.9 ± 51.8^ab^	895.2 ± 49.7^ab^	726.4 ± 13.0^b^

*Note:* Fermentation was carried out for 72 h at 30°C. BSG incubated under the same conditions, except for the use of starters, was used as the control (Unstarted‐BSG). Data represents the mean ± standard deviation of two biological replicates (each analysed in technical triplicate). ^a–c^means within the row with different letters are significantly different (*p* < 0.05).

Abbreviation: n.d., not detected.

### 3.6 Phenolic Compounds Profiling

Forty analytical standards were used to identify and quantify the main free phenolic compounds of Raw‐, Unstarted‐ and Fermented‐BSG samples using LC‐HRMS. Among these, 18 compounds were detected, with catechin, vanillin, procyanidin B3, ferulic acid, *p*‐coumaric acid, procyanidin B1 and protocatechuic acid being the most abundant in Raw‐BSG, ranked in descending order (Table 2). Our experimental conditions unveiled significant (*p* < 0.05) fluctuations in the phenolic compounds profile of Raw‐BSG attributed to the interplay between autochthonous microbiota and the inoculum of cultures during incubation. Fermentation led to complete hydrolysis of *p*‐coumaric acid and ferulic acid, except for limited concentrations found in GY1‐BSG for ferulic acid. Likewise, significant (*p* < 0.05) metabolism of protocatechuic and caffeic acids was observed, with the highest values in GY1‐BSG. Vanillin and syringaldehyde witnessed a significant (*p* < 0.05) reduction, with varying levels among the Fermented‐BSG samples. FLAB and yeast showed contrasting effects on gallic acid metabolism, with notable (*p* < 0.05) depletion observed in PL22‐BSG, while significant release occurred in GY1‐BSG. Procyanidin B1 and B3 concentrations experienced notable (*p* < 0.05) decreases only in Unstarted‐BSG and PL22‐BSG, whereas GY1‐BSG showed comparable (*p* > 0.05) levels to those in Raw‐BSG. Catechol was undetected in Raw‐BSG, but generated during fermentation, with the lowest levels in Unstarted‐BSG, followed by PL22‐BSG, and the highest values in GY1‐BSG. Phloretin also showed an upward trend in fermented samples, particularly pronounced (*p* < 0.05) in PL22‐BSG. On the other hand, GY1‐BSG showed significantly (*p* < 0.05) higher levels of kampferol, isorhamnetin and gallocatechin compared to Raw‐ and Unstarted‐BSG samples. Furthermore, exclusive detection of quercetin and naringenin was observed only in BSG fermented with *W. anomalus* GY1 (Table 2).

**TABLE 2:** Quantification of free phenolic compounds (μg/g DW) by LC‐HRMS in methanol water soluble extract (MWSE) obtained from raw brewer's spent grain (Raw‐BSG) and BSG fermented with *Fructobacillus fructosus* PL22 (PL22‐BSG) and *Wickerhamomyces anomalus* GY1 (GY1‐BSG).

Compounds	Raw‐BSG	Unstarted‐BSG	PL22‐BSG	GY1‐BSG
Gallic acid	99.4 ± 1.5^b^	94.8 ± 3.7^b^	72.0 ± 1.5^c^	125.9 ± 2.1^a^
Protocatechuic acid	1176.5 ± 151.3^a^	317.5 ± 30.8^c^	273.2 ± 2.5^c^	807.8 ± 126.9^b^
Procyanidin B1	1444.1 ± 84.7^a^	826.6 ± 28.3^b^	765.6 ± 26.4^b^	1541.0 ± 83.8^a^
Gallocatechin	124.5 ± 8.4^b^	127.2 ± 4.3^b^	125.9 ± 2.8^b^	158.0 ± 12.2^a^
Catechol	n.d.	254.5 ± 19.3^c^	357.5 ± 19.0^b^	529.2 ± 53.0^a^
Procyanidin B3	3855.1 ± 289.1^a^	1514.8 ± 272.0^b^	1155.8 ± 34.7^b^	3645.1 ± 565.7^a^
Caffeic acid	95.2 ± 9.7^a^	45.9 ± 2.4^c^	44.6 ± 1.4^c^	79.6 ± 5.7^b^
Catechin	7019.2 ± 975.2^a^	6142.8 ± 44.4^a^	3358.7 ± 13.6^b^	6922.0 ± 667.6^a^
*p*‐coumaric acid	2238.9 ± 347.7	n.d.	n.d.	n.d.
Vanillin	6390.7 ± 486.4^a^	714.4 ± 19.7^bc^	1090.5 ± 54.0^b^	358.6 ± 26.4^c^
Syringaldehyde	681.7 ± 16.9^a^	30.4 ± 11.5^c^	100.0 ± 2.8^b^	n.d.
Ferulic acid	2929.3 ± 590.0^a^	n.d.	n.d.	104.8 ± 25.4^b^
Quercetin	n.d.	n.d.	n.d.	39.5 ± 2.6
Naringenin	n.d.	n.d.	n.d.	176.0 ± 19.6
Phloretin	2.5 ± 1.5^d^	11.1 ± 0.3^c^	15.0 ± 0.7^a^	12.8 ± 0.6^b^
Genistein	222.5 ± 39.9^a^	55.2 ± 29.2^b^	200.3 ± 38.5^a^	n.d.
Kaempferol	312.4 ± 12.2^b^	295.8 ± 12.9^b^	323.1 ± 15.4^b^	391.4 ± 22.2^a^
Isorhamnetin	226.2 ± 39.8^b^	181.1 ± 34.0^b^	268.3 ± 39.1^ab^	367.0 ± 50.8^a^

*Note:* Fermentation was carried out for 72 h at 30°C. BSG incubated under the same conditions, except for the use of starters, was used as the control (Unstarted‐BSG). Data represent the mean ± standard deviation of two biological replicates (each analysed in technical triplicate). ^a–d^means within the row with different letters are significantly different (*p* < 0.05).

Abbreviation: n.d., not detected.

### 3.7 Antifungal and Antioxidant Activities

**FIGURE 4:**
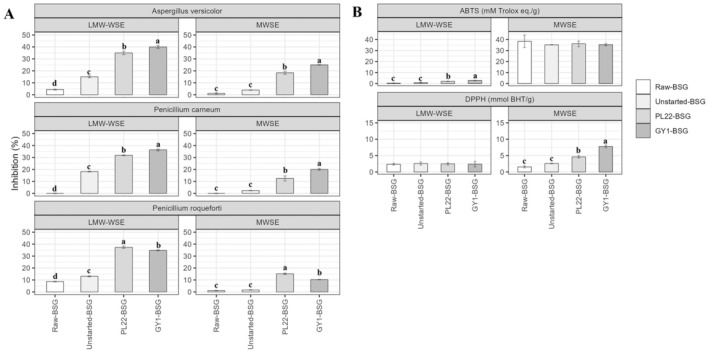
In vitro hyphal radial growth inhibition (%) against *Penicillium roqueforti* DPPMA1, *Aspergillus versicolor* CBS 117286 and *Penicillium carneum* CBS112297 (A) and ABTS (mM Trolox eq./g) and DPPH (mmol BHT/g) radical scavenging activity (B) of low molecular weight water soluble extracts (LMW‐WSE) and methanol–water soluble extracts (MWSE) obtained from raw brewer's spent grain (Raw‐BSG) and BSG fermented with *Fructobacillus fructosus* PL22 (PL22‐BSG) and *Wickerhamomyces anomalus* GY1 (GY1‐BSG). Fermentation was carried out for 72 h at 30°C. BSG incubated under the same conditions, except for the use of starters, was used as the control (Unstarted‐BSG). Data represent the mean ± standard deviation of two biological replicates (each analysed in technical triplicate). Bars with different superscript letters differ significantly (*p* < 0.05). *P. roqueforti* DPPMA1, *A. versicolor* CBS 117286 and *P. carneum* CBS112297 without treatments were used as controls. Bars with different superscript letters differ significantly (*p* < 0.05).

Antifungal activity of LMW‐WSE and MWSE from Raw‐, Unstarted‐ and Fermented‐BSG samples was assessed against three food spoilage‐related indicators (Figure 4A). Besides the extract type, the most pronounced inhibition (*p* < 0.05) against *Aspergillus versicolor* and *Penicillium carneum* was found in GY1‐BSG, followed by PL22‐BSG. Conversely, PL22‐BSG had the highest inhibitory activity against *Penicillium roqueforti*, followed by GY1‐BSG. Overall, Unstarted‐BSG showed higher values (*p* < 0.05) than Raw‐BSG when using LMW‐WSE, whereas no difference (*p* > 0.05) was observed with MWSE (Figure 4A).

The same extracts were used to evaluate the antioxidant activity through ABTS (2,20‐azino‐bis(3‐ethylbenzothiazoline‐6‐sulphonic acid)) and DPPH (1,1‐diphenyl‐2‐picrylhydrazyl radical) assays (Figure 4B). Compared to that in Raw‐BSG, the ABTS scavenging activity showed a significant (*p* < 0.05) increase only in LMW‐WSE from fermented samples, especially in GY1‐BSG. On the contrary, the DPPH scavenging activity was significantly (*p* < 0.05) enhanced in started samples only when using MWSE (Figure 4B).

### 3.8 Effect of Fermented BSG on Human Keratinocytes Cell Line Viability

No cytotoxic effect was found across all analysed conditions, except for Unstarted‐BSG at 10 mg/mL after 72 h of treatment, which reduced the cell viability to 64.09% ± 18.94%.

### 3.9 Effect on the Migration After Scratching

**FIGURE 5:**
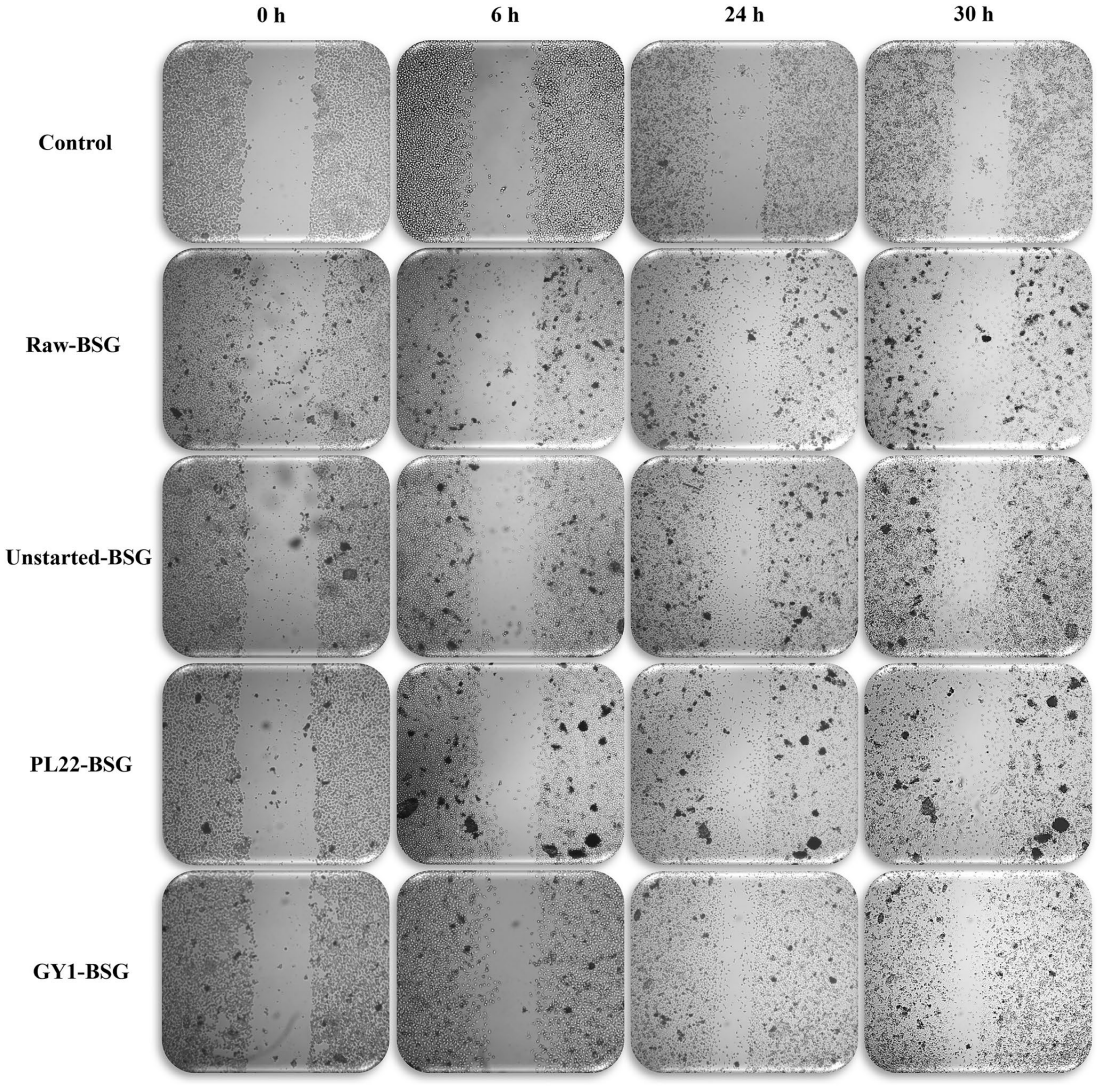
Representative images of the effect of raw brewer's spent grain (Raw‐BSG) and BSG fermented with *Fructobacillus fructosus* PL22 (PL22‐BSG) and *Wickerhamomyces anomalus* GY1 (GY1‐BSG) on the migration of human keratinocyte NCTC 2544 cells. Fermentation was carried out for 72 h at 30°C. BSG incubated under the same conditions, except for the use of starters, was used as the control (Unstarted‐BSG). Sub‐confluent monolayers of NCTC 2544 cells were scratched with a sterile P200 pipette tip and treated (at 37°C for 30 h, under 5% of CO_2_), with basal serum‐free medium alone (control); 0.5 mg/mL of Raw‐BSG; 0.5 mg/mL of Unstarted‐BSG; 0.5 mg/mL of PL22‐BSG; or 0.5 mg/mL of GY1‐BSG.

The in vitro scratch assay was used to determine the effect of BSG on proliferation and wound healing capacity of NCTC 2544 cells. Compared to that in the control (basal serum free medium alone), the concentration of 0.5 mg/mL of the BSG samples accelerated the cell migration toward the open area more effectively than 0.1 mg/mL (Table S1). This effect appeared 6 h after scratching and showed a progressive increase up to 30 h (Figure 5). A positive effect on cell migration/proliferation was also found using hyaluronic acid (Table S1). Images were subjected to statistical analyses for highlighting the differences between treatments. The percentage of open scratch area of NCTC 2544 cells subjected to all treatments showed reductions compared to that in the control at all time points. This effect was especially pronounced (*p* < 0.05) in cells treated with GY1‐BSG, in which the percentage of open area after 6, 24 and 30 h of treatment was ca. 69.64%, 63.40% and 50.03%, respectively.

### 3.10 Transcriptional Regulation of *FLG* Gene

**FIGURE 6:**
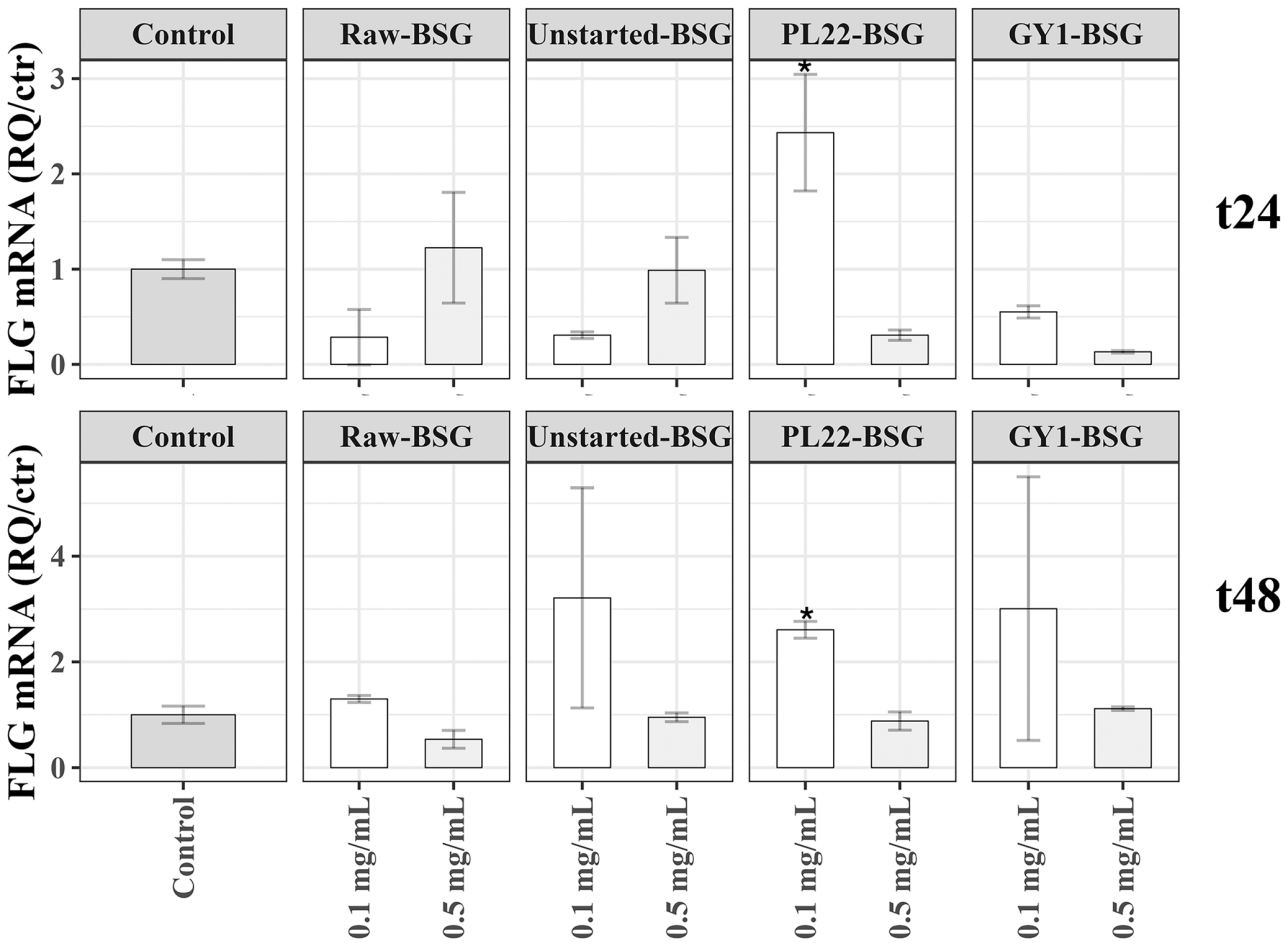
Filaggrin (*FLG*) gene expression in human keratinocyte cell line NCTC 2544 through quantitative reverse transcription‐polymerase chain reaction (qRT‐PCR). Cells were treated for 24 (t24) and 48 h (t48) with samples of raw brewer's spent grain (Raw‐BSG), Unstarted‐BSG, or BSG fermented with *Fructobacillus fructosus* PL22 (PL22‐BSG) and *Wickerhamomyces anomalus* GY1 (GY1‐BSG). Fermentation was carried out for 72 h at 30°C. Untreated NCTC 2544 cells were used as control. Data represent the mean ± standard deviation of two biological replicates (each analysed in technical duplicate). Asterisk indicates a significant difference (*p* < 0.05) with respect to the control. Details about the sample preparation are described in the material and methods section.

The expression of the *FLG* gene in NCTC 2544 cells was determined following treatment with Raw‐, Unstarted‐ and Fermented‐BSG samples. PL22‐BSG at 0.1 mg/mL significantly upregulated *FLG* gene expression at both 24 and 48 h (*p* < 0.05) (Figure 6). Other BSG samples also enhanced *FLG* gene expression at 48 h, though these increases were not statistically significant (*p* > 0.05).

## 4 Discussion

In a world challenged by waste production and the need for sustainable and innovative solutions, effective measurements to harness the potential of underutilised resources are highly required. Through a tailored fermentation and targeted metabolism of FLAB and yeast, we highlighted significant modifications in the bioactive compounds profile of the BSG by‐product, conferring enhanced bio‐functional properties for potential sustainable applications in food, pharmaceuticals and especially cosmetic industries.

The high moisture content and rich nutrient composition of BSG provide an optimal niche for microbial proliferation, with mesophilic and thermophilic bacteria, alongside yeasts and moulds (4–5 Log CFU/g), representing the predominant indigenous microbiota (Robertson et al. 2010). These microorganisms may drive spontaneous fermentation by acting as spoilage agents and accelerating the degradation of BSG components (Bianco et al. 2020). To counter these challenges, dominate indigenous microbiota and ensure a controlled and resilient fermentation, non‐conventional *F. fructosus* PL22 and *W. anomalus* GY1, renowned for their antimicrobial, antifungal and probiotic traits (Coda et al. 2011; Patil et al. 2020; Tonini et al. 2024), were investigated as starter cultures. After fermentation, although these microorganisms effectively dominated the BSG environment, they were unable to inhibit the growth of indigenous microorganisms (Robertson et al. 2010), allowing LAB and yeasts to coexist in all fermented samples as revealed by RAPD‐RCR profiling. This unravelled microbial complexity hindered the distinction between the carbohydrate metabolism pathways specific to LAB and yeasts, as all samples showed a similar reduction in free sugars. Nevertheless, the impact of the starter‐assisted fermentation became evident through the production of key microbial metabolites (Endo et al. 2018; Wang et al. 2020; Padilla et al. 2018). Specifically, *F. fructosus* induced the highest levels of lactic and acetic acids (Mohamed et al. 2023), correlating with the most pronounced acidification. Unlike its fermentation of apple blossoms (Tonini et al. 2024), no mannitol was found in the Fermented‐BSG, likely due to the substrate composition affecting its metabolic activity (Zannini et al. 2022). This suggests potential mannitol degradation in BSG, possibly mediated by a mannitol‐specific PTS system and mannitol‐1‐phosphate‐dehydrogenase (Neves et al. 2002; Wisselink et al. 2005). *Wickerhamomyces anomalus* predominantly released ethanol and was found in Unstarted‐BSG due to the indigenous yeasts.

Two distinct patterns in protein metabolism were revealed during BSG fermentation. In line with previous studies, the first involved an increase in protein content (Knez et al. 2023; Anyiam et al. 2023), likely linked to the ability of *F. fructosus* to synthesise single‐cell protein (Patel et al. 2019), an important element in developing functional ingredients. The second pattern showed no alteration in protein content, highlighting the importance of peptide profiling throughout the fermentation to comprehensively understand these dynamics. Fermentation with *F. fructosus* PL22 and *W. anomalus* GY1 generated peptides, whose profile was microbial‐dependent. The bioactivity of peptides released during fermentation is frequently found (López‐García et al. 2022; Rizwan et al. 2023), especially exerted by low molecular weight peptides, which are more bioactive due to their higher absorption and interaction with biological systems (Garcés‐Rimón et al. 2022). The HPLC‐HRMS analysis showed that fermentation reduced the peptide abundance but increased their diversity, with Started‐BSG producing the highest number of unique peptides. Nevertheless, most did not match known bioactive peptides because the BIOPEP UWM database, used for screening, predominantly contains sequences from animal proteins rather than plant proteins (Tonini et al. 2024). Therefore, we cannot exclude other potential bioactivities. The two exceptions were the ACE inhibitory peptide FTTQ, present in all BSG samples, particularly Raw‐BSG, supporting its value as a low‐cost protein source to extract bioactive peptides for food applications (Abeynayake et al. 2022), and the antioxidant peptide SVNVPLY, found only in the BSG fermented with the FLAB. Both peptides have been previously identified in hydrolysates from *Phaseolus vulgaris* (Mojica et al. 2015) and potent antioxidant fractions of barley protein hydrolysate (Bamdad and Chen 2013; Eckert et al. 2016).

Given the role of amino acids in amplifying bioactivity (Aursuwanna et al. 2022; Akbarian et al. 2022), the influence of started fermentation in altering their composition is noteworthy. The observed reduction of various amino acids during fermentation reflected the metabolic strategies of microorganisms that utilise these substrates for energy production, redox balance and biosynthetic processes (Méndez et al. 2022; Wang et al. 2021). In contrast, Ala, Cys and GABA increased only when BSG was fermented with *W. anomalus* GY1. Alanine can enhance antioxidant activity and bioactivity through glutathione synthesis and free radical scavenging, while Cys can mitigate hydroxyl radical formation (Meucci and Mele 1997; Dyachenko and Bel'skaya 2023). The predominance of prolamins in BSG (Chin et al. 2022) accounted for the high Pro levels relative to other amino acids. Proline functions as a crucial signalling molecule that regulates antioxidative reactions and immune responses (Wu et al. 2011), and notably, its concentration was further enhanced only through the fermentation process in PL22‐BSG.

Phenolic compounds in plant‐based products provide various health benefits (Zhao et al. 2021), positioning BSG as a sustainable source rich in hydroxycinnamic acids and procyanidins (Panzella et al. 2020; Macias‐Garbett et al. 2021). Distinguishing phenolic alterations between spontaneous and starter fermentations was challenging, as most compounds exhibited similar trends, albeit with varying intensities, with only a few showing notable differentiation due to starters. A marked reduction in protocatechuic acid accompanied by an increase in catechol was predominantly observed in GY1‐BSG. Catechol can act both as an antioxidant, preventing lipid peroxidation, and as a pro‐oxidant damaging macromolecules, such as DNA and proteins (Schweigert et al. 2001). Likewise, the release of aglycones, including quercetin and naringenin, known for their anti‐obesity, antidiabetic and antifibrotic properties (Jadeja and Devkar 2014), was exclusive to GY1‐BSG, which also showed elevated levels of other health‐promoting compounds, such as gallic acid, gallocatechin, kaempferol and isorhamnetin (Xue et al. 2023). Conversely, phloretin, noted for its anticancer, antioxidant and anti‐inflammatory effects (Fan et al. 2022), was found in higher concentrations in all the fermented samples, especially in PL22‐BSG. While the mechanisms behind these transformations remain to be elucidated, possibly requiring transcriptomic or enzymatic data, our findings highlight the impact of microbial fermentation on the phenolic profile of BSG and suggest its potential for the development of functional ingredients.

The bioactive modifications during BSG fermentation achieved functional properties, including antifungal and antioxidant activities (Sharma et al. 2019). Although no specific antifungal bioactive peptides were detected, the generation of new peptides, mainly in Started‐BSG, may account for their antifungal potential. Inhibition effects were revealed only in methanol extracts from FLAB‐ and yeast‐fermented BSG. The release of several phenolics, such as gallic acid, gallocatechin, catechol, quercetin, naringenin and kaempferol, likely contributed to the observed inhibition in GY1‐BSG (Park et al. 2011; Nguyen and Bhattacharya 2022; Rocha et al. 2019), but explaining such inhibition in PL22‐BSG was more challenging due to its phenolic profile being similar to Unstarted‐BSG. Catechol and phloretin levels were elevated, while gallic acid and catechin were reduced, which may have facilitated the production of other functional metabolites (Fang et al. 2019) not detected by our targeted analytical approach. Overall, our findings reaffirmed the contributory role of our non‐conventional microbial species in the biosynthesis of antifungal compounds, consistent with previous observations across diverse substrates, including sourdough (Acín Albiac et al. 2020), pollen (Di Cagno et al. 2019) and apple flowers (Tonini et al. 2024). The microbial fermentation of BSG, especially that driven by the yeast, exerted antioxidant activity as demonstrated by the efficacy against ABTS, indicating that newly generated peptides and the release of Ala and Cys in GY1‐BSG contributed to this effect. The antioxidant peptide identified in PL22‐BSG did not directly correspond to the highest observed increase in antioxidant capacity, supporting the hypothesis that unidentified bioactive peptides may also be involved. In contrast, methanol extracts from Started‐BSG effectively scavenged only DPPH radicals, indicating that the phenolic changes may also mediate antioxidant effects by favouring hydrogen donation over electron transfer (Ivanova et al. 2020).

While bioactive compounds can protect the skin and address dermatological concerns (Turcov et al. 2023), empirical evidence in this field remains scarce, and little is known about the effects of direct exposure to fermented BSG on the human skin layer. As in vitro assays are only predictive of in vivo functional activity, and direct testing on animals or humans is not straightforward (Verni et al. 2019), a cellular model was employed in our screening. A human keratinocyte cell line, the primary barrier of the human body and a useful target for cytotoxic studies (Cappelli et al. 2021), was exposed to fermented BSG and afterwards, the MTT assay assessed its effects on cell viability. None of our experimental conditions after 24 or 48 h of incubation exerted a cytotoxic effect. Generally, cutaneous repair is a multifaceted wound healing process which involves several types of cells and requires blood clotting, inflammation of the involved area, and generation and remodelling of a new tissue (Ding et al. 2023; Pinto et al. 2011). Proliferation and migration of cells are essential mechanisms driving the wound healing process. Consequently, exploring novel agents that stimulate tissue regeneration and promote wound healing is important for advancing regenerative therapies. Existing literature established a strong association between enhanced wound healing and the antibacterial effects of phenolic compounds (Abate et al. 2021), explaining the reduction of scratch area in GY1‐BSG, attributed to its higher levels of gallic acid, quercetin and naringenin, which are vital for regulating the wound healing process (Trinh et al. 2022). Specifically, quercetin promotes fibroblast migration and proliferation while reducing inflammation through antioxidant activities (Kant et al. 2020; Mi et al. 2022). Gallic acid mitigates oxidative stress, accelerates keratinocyte migration and activates wound healing factors (Yang et al. 2016). Naringenin exerts anti‐inflammatory and antioxidant effects, influencing inflammation and proliferation in wound healing (Trinh et al. 2022). Being a protein primarily associated with the formation and maintenance of the skin barrier, filaggrin has also been implicated in wound healing processes through its role in keratinocyte differentiation and proliferation (Kurokawa et al. 2006). Aberrations in filaggrin expression may affect the integrity and function of the epidermal barrier, leading to impaired wound healing (G. Yang et al. 2020). Therefore, understanding the relationship between filaggrin and wound healing could provide insights into developing therapeutic strategies for enhancing skin repair and mitigating chronic wound conditions. Based on our findings, the expression of the *FLG* gene was upregulated by the treatment of keratinocyte cells with PL22‐BSG, suggesting an increase in the activity of the filaggrin protein and therefore improving the epidermal skin barrier. Similarly, *FLG* upregulation was observed in HaCaT keratinocytes when treated with cell‐free supernatants from other probiotic LAB strains (Lee et al. 2023). Despite the enhanced wound healing observed in GY1‐BSG, the lack of *FLG* gene upregulation may suggest the involvement of alternative mechanisms, potentially linked to pathways governing cell proliferation, migration, or extracellular matrix remodelling, such as the TGF‐β/SMAD signalling (Mokoena et al. 2018).

## 5 Conclusion

The metabolic potentiality of non‐conventional microbial species, *F. fructosus* PL22 and *W. anomalus* GY1, in harnessing the latent bioactive reservoir of BSG was demonstrated. Despite a reduction in LMW peptides abundance, both species promoted the generation of unique peptides, with only PL22‐BSG producing one previously identified peptide with antioxidant properties. The GY1‐BSG exclusively facilitated the release of amino acids and key phenolics. These metabolic shifts across different species correlated with improved antifungal, antioxidant and skin‐protective properties. Our targeted approach sheds light on the mechanisms behind changes in the profile of specific compound classes driven by the starters during the controlled fermentation of BSG. While this strategy yielded valuable insights, incorporating metagenomic analysis in future studies would offer a more holistic view of microbial dynamics and their functional roles throughout BSG fermentation. Additionally, untargeted characterisation of unfractionated extracts could uncover synergistic interactions among different classes of molecules, as well as matrix effects that may underlie the observed bioactivities.

## Author Contributions

**Alessandro Stringari:** investigation, methodology, formal analysis, writing – original draft, data curation. **Ali Zein Alabiden Tlais:** conceptualization, investigation, methodology, validation, formal analysis, writing – review and editing, data curation. **Stefano Tonini:** investigation, methodology, formal analysis, data curation. **Daniela Pinto:** investigation, formal analysis, data curation. **Giorgia Mondadori:** investigation, data curation, formal analysis. **Pasquale Filannino:** writing – review and editing, validation. **Raffaella Di Cagno:** supervision, writing – review and editing, conceptualization. **Marco Gobbetti:** funding acquisition, project administration, supervision.

## Conflicts of Interest

The authors declare no conflicts of interest.

## Supporting information


Figure S1.

Figure S2.

Figure S3.

Table S1.

## Data Availability

The data that support the findings of this study are available from the corresponding author upon reasonable request.
