# Supplementary material for: Revitalising Brewers' Spent Grains and Enriching With Biogenic Compounds Through the Fermentation of Fructophilic Lactic Acid Bacteria and Yeasts
Source: Microb Biotechnol. 2025 Jun 9;18(6):e70171. doi: 10.1111/1751-7915.70171 (PMC12149443; doi:10.1111/1751-7915.70171)

**Figure S1.** Representative Random Amplified Polymorphic DNA-Polymerase Chain Reaction (RAPD-PCR) patterns of starters and isolates of presumptive lactic acid bacteria (A) and yeasts (B) from raw brewer’s spent grain (Raw-BSG) and BSG fermented with *Fructobacillus fructosus* PL22 (PL22-BSG) and *Wickerhamomyces anomalus* GY1 (GY1-BSG). Fermentation was carried out for 72 h at 30 °C. BSG incubated under the same conditions, except for the use of starters, was used as the control (Unstarted-BSG). DNA molecular size standards (10000-250 bp) (M); *F. fructosus* PL22 (R1); *W. anomalus* GY1 (R2). Primer P4 for lactic acid bacteria and mM13 for yeasts were used for RAPD-PCR analysis.


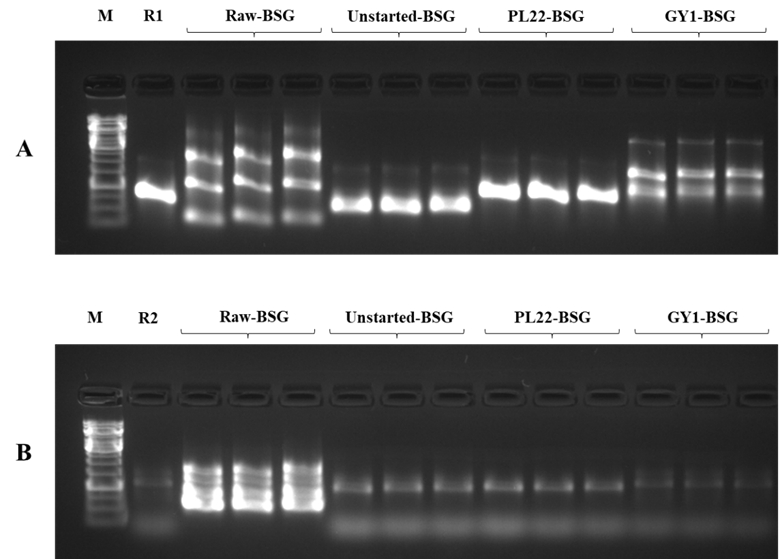

Supplement: Supplementary file 1 — Figure S1. [file MBT2-18-e70171-s004.docx]
