# Supplementary material for: Revitalising Brewers' Spent Grains and Enriching With Biogenic Compounds Through the Fermentation of Fructophilic Lactic Acid Bacteria and Yeasts
Source: Microb Biotechnol. 2025 Jun 9;18(6):e70171. doi: 10.1111/1751-7915.70171 (PMC12149443; doi:10.1111/1751-7915.70171)

**Figure S3.** Peptides profile obtained through RP-FPLC (detector 240 mm) chromatograms in raw brewer’s spent grain (Raw-BSG) and BSG fermented with *Fructobacillus fructosus* PL22 (PL22-BSG) and *Wickerhamomyces anomalus* GY1 (GY1-BSG). Fermentation was carried out for 72 h at 30 °C. BSG incubated under the same conditions, except for the use of starters, was used as the control (Unstarted-BSG).


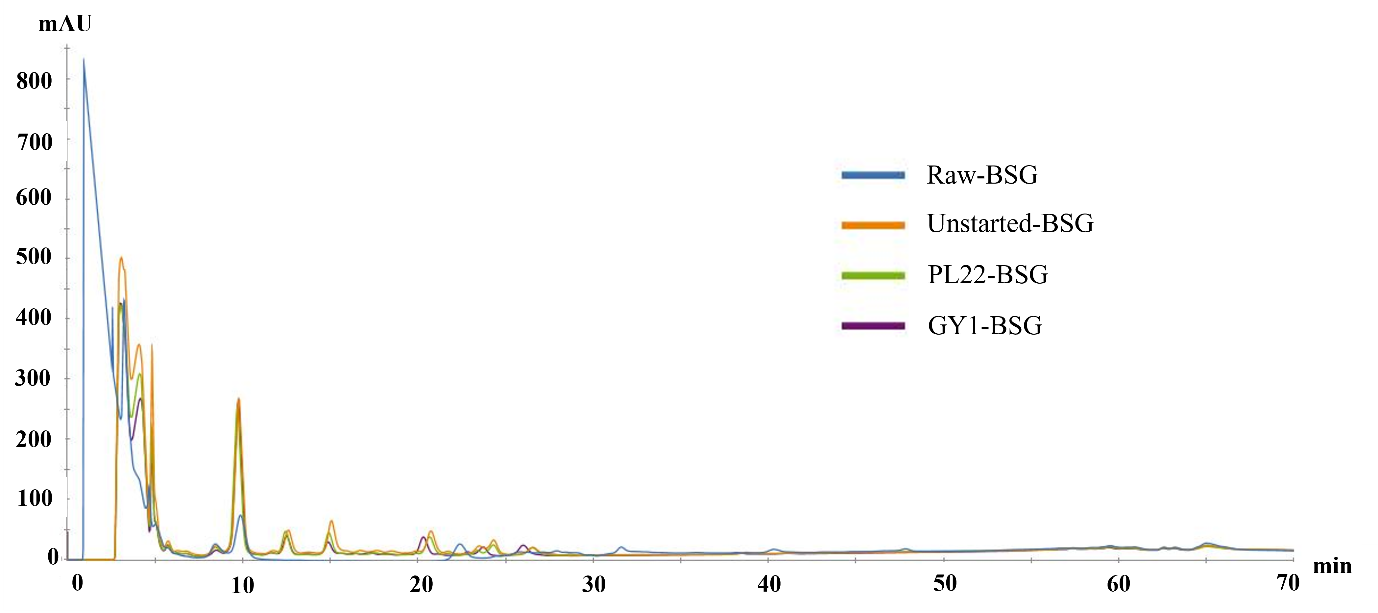

Supplement: Supplementary file 3 — Figure S3. [file MBT2-18-e70171-s002.docx]
