# Supplementary material for: Revitalising Brewers' Spent Grains and Enriching With Biogenic Compounds Through the Fermentation of Fructophilic Lactic Acid Bacteria and Yeasts
Source: Microb Biotechnol. 2025 Jun 9;18(6):e70171. doi: 10.1111/1751-7915.70171 (PMC12149443; doi:10.1111/1751-7915.70171)
Supplement: Supplementary file 4 — Table S1. [file MBT2-18-e70171-s001.docx]

**Table S1.** Effect (% of the control) of raw brewer’s spent grain (Raw-BSG), Unstarted-BSG, BSG fermented with *Fructobacillus fructosus* PL22 (PL22-BSG) and *Wickerhamomyces anomalus* GY1 (GY1-BSG), or hyaluronic acid on migration of human keratinocyte NCTC 2544 cells. Fermentation was carried out for 72 h at 30 °C. Sub-confluent monolayers of NCTC 2544 cells were scratched with a sterile P200 pipette tip and treated (at 37 °C for 30 h, under 5% of CO_2_), with: basal serum free medium alone (control); 0.5 and 0.1 mg/mL of Raw-BSG; 0.5 and 0.1 mg/mL of Unstarted-BSG; 0.5 and 0.1 mg/mL of PL22-BSG; 0.5 and 0.1 mg/mL of GY1-BSG; or 2 µg/mL of hyaluronic acid.

| **Samples** | **6h** | **24h** | **30h** |
| --- | --- | --- | --- |
| Control | 100.00 ± 5.14 | 100.00 ± 13.30 | 100.00 ± 13.98 |
| Raw-BSG 0.5 mg/mL | 99.19 ± 4.24 | 84.89 ± 3.63 | 85.97 ± 2.98 |
| Raw-BSG 0.1 mg/mL | 102.61 ± 3.29 | 104.41 ± 4.30 | 106.61 ± 2.08 |
| Unstarted-BSG 0.5 mg/mL | 93.54 ± 2.61 | 76.12 ± 10.81 | 70.13 ± 7.17 |
| Unstarted-BSG 0.1 mg/mL | 94.72 ± 5.17 | 91.58 ± 10.00 | 83.87 ± 16.16 |
| PL22-BSG 0.5 mg/mL | 97.29 ± 5.65 | 93.74 ± 5.37 | 84.19 ± 8.93 |
| PL22-BSG 0.1 mg/mL | 97.79 ± 5.67 | 103.87 ± 4.42 | 101.93 ± 2.50 |
| GY1-BSG 0.5 mg/mL | 69.64 ± 12.48* | 63.40 ± 3.29* | 50.03 ± 7.72* |
| GY1-BSG 0.1 mg/mL | 90.61 ± 8.29 | 84.23 ± 9.33 | 81.64 ± 14.12 |
| Hyaluronic acid 2 µg/mL | 101.46 ± 0.00 | 91.17 ± 0.66 | 87.40 ± 4.80 |

* indicates significant differences (P < 0.05) compared to the Control
